# High keratinase and other types of hydrolase activity of the new strain of *Bacillus paralicheniformis*

**DOI:** 10.1371/journal.pone.0312679

**Published:** 2024-10-25

**Authors:** Saniya Aktayeva, Bekbolat Khassenov

**Affiliations:** 1 Laboratory for Genetics and Biochemistry of Microorganisms, National Center for Biotechnology, Astana, Kazakhstan; 2 Faculty of Natural Sciences, L.N. Gumilyev Eurasian National University, Astana, Kazakhstan; University of Hawai’i at Manoa, UNITED STATES OF AMERICA

## Abstract

Keratinases, a subclass of proteases, are used to degrade keratin thereby forming peptones and free amino acids. *Bacillus paralicheniformis* strain T7 was isolated from soil and exhibited high keratinase, protease, collagenase, amylase, xylanase, lipase, and phosphatase activities. Keratinases of the strain showed maximum activity at 70°C and pH 9.0 as well as high thermal stability. A mass-spectrometric analysis identified seven peptidases with molecular masses of 26.8–154.8 kDa in the secretory proteome. These peptidases are members of S8 and S41 serine peptidase families and of M14, M42, and M55 metallopeptidase families. Additionally, α-amylase (55.2 kDa), alkaline phosphatase (59.8 kDa), and esterase (26.8 kDa) were detected. The strong keratinolytic properties of the strain were confirmed by degradation of chicken and goose feathers, which got completely hydrolyzed within 4 days. Submerged fermentation by strain *B*. *paralicheniformis* T7 was carried out in a pilot bioreactor, where the highest keratinase production was noted after 19 h of cultivation. After the fermentation, in the culture fluid, the keratinase activity toward keratin azure was 63.6 ± 5.8 U/mL. The protease activity against azocasein was 715.7 ± 40.2 U/mL. The possibility of obtaining enzyme preparations in liquid and powder form was demonstrated, and their comparative characteristics are given. In the concentrate, the keratinase, protease, α-amylase, phosphatase, and esterase/lipase activities were 2,656.7 ± 170.4, 29,886.7 ± 642.9, 176.1 ± 16.3, 23.9 ± 1.8, and 510.9 ± 12.2 U/mL, respectively. In the lyophilizate, these activities were 57,733.3 ± 8,911.4, 567,066.7 ± 4,822.2, 2,823.0 ± 266.8, 364.2 ± 74.8, and 17,618.0 ± 610.3 U/g, respectively. In the preparation obtained by air flow drying at 55°C, these activities were 53,466.7 ± 757.2, 585,333.3 ± 4,277.1, 2,395.8 ± 893.7, 416.7 ± 52.4, and 15,328.1 ± 528.6 U/g, respectively. The results show high potential of *B*. *paralicheniformis* strain T7 as a producer of keratinases and other enzymes for applications in agricultural raw materials and technologies for processing of keratin-containing animal waste.

## Introduction

Poultry production is the most developed sector of the livestock industry. Modern poultry meat–processing technology allows for the utilization of almost all poultry carcasses, but poultry feathers are not subjected to any processing. At the same time, feathers are composed of proteins at >90%, mainly keratin, and other insoluble ones [1]. Being densely packed in the β-sheet polypeptide chain, abundantly cross-linked by disulfide bonds, and stabilized by noncovalent interactions, keratin is one of the most robust proteins [2]. According to their secondary structure, proteins of the keratin family are categorized into two groups: α-keratins, which have a conformation in the form of tight coils around the long axis of the molecule’s α-helix (the basis of hair, wool, horns, claws, and hooves of mammals), and β-zigzag polypeptide chains: β-sheets (the basis of claws, scales of lizards, turtle shells, feathers, beaks, and claws of birds) [3]. Physical and chemical methods of processing of feathers lead to the formation of some non-nutritional amino acids such as lysinoalanine and lanthionine and reduce the amino acid content and digestibility of feather meal [4]. Enzymatic hydrolysis maximally preserves nutritional value of products and significantly increases their solubility and digestibility. Nonetheless, keratins are highly resistant to the action of proteolytic enzymes because of the abundance of disulfide and hydrogen bonds and hydrophobic interactions [5]. Keratin is difficult to cleave by known proteases like pepsin, trypsin, and papain [6]; special proteases having keratinolytic activity, named keratinases, are required for its hydrolysis. Recently, a large number of studies were focused on keratinases from *Bacillus* [7–11]. High biotechnological potential of bacterial keratinases has attracted much attention due to their numerous applications in industry for the development of environmentally friendly processes. In practice, keratinases have an advantage over proteases because the former can make feathers a cheap source of feed amino acids and peptides [12].

Microbial keratinases can be employed for enzymatic hydrolysis of keratin-containing substrates [13], as a feed additive [14], and for effective waste management [15]. For example, keratinases from *Bacillus paralicheniformis* MKU3 have shown promise in hair removal from hides [16], while surfactant-stable keratinase from *Bacillus cereus* YQ15 can serve as a detergent additive for removing blood stains [17]. Keratinases from *Ochrobactrum intermedium* NKIS 1 have potential applications in the biodegradation of chicken feathers and can add value to poultry waste [18]. Additionally, keratinases are essential for the preparation of animal nutrients and have applications in the pharmaceutical and biomedical industries [19]. *Bacillus* species are also effective producers of amylases [5], xylanases [20, 21], lipases [22], and phosphatases [23].

Information on strains that hydrolyze natural polymers is regularly updated; however, it is becoming clear that the biodegradation of keratin involves not just a single protease but a set of enzymes. Therefore, there is a need for research that includes the identification and characterization of a secreted enzyme cocktail. This work describes keratinases of *Bacillus paralicheniformis* strain T7, isolated from soil and showing high keratinase activity. Their qualitative and quantitative characteristics were investigated by biochemical, zymographic, mass spectrometric, proteomic, and spectrophotometric methods. Chicken and goose feathers were hydrolyzed by the strain, and the hydrolysis products were studied by scanning electron microscopy (SEM). Correlations between the genome of the strain, its proteome, and its biochemical activity were evaluated. It was shown that submerged fermentation by *B*. *paralicheniformis* strain T7 in a bioreactor could be used to obtain a keratinase preparation. Additionally, other types of hydrolase activity in this preparation were investigated: protease, α-amylase, phosphatase, and esterase activities.

## Materials and methods

### Reagents and media

Ovalbumin was acquired from MP Biomedicals (USA), whereas bovine serum albumin (BSA), hemoglobin, and casein sodium salt from Sigma (St. Louis, MO, USA). Phenylmethylsulphonyl fluoride (PMSF), Pepstatin A, ethylenediaminetetraacetic acid (EDTA), E64 and chemical reagents were purchased from Sigma and AppliChem (Darmstadt, Germany), and gelatin and nutrient ingredients from Titan (India).

The following liquid nutrient media were employed for cultivation: nutrient broth and feather broth (0.3 g/L NaH_2_PO_4_, 0.35 g/L Na_2_HPO_4_, and 7.5 g/L feather, pH 7.0). To isolate and test the strain, different types of agar-based media were used. These included nutrient agar (Nutrient Broth with 1.5% of agar), skim milk agar (2% of skim milk, 0.1% of NaCl, 1% of tryptone, and 1% of agar), gelatin agar (0.4% of peptone, 0.1% of yeast extract, 1.5% of gelatin, and 1.5% of agar), and feather agar (1.7 g/L NaH_2_PO_4_, 0.35 g/L feather powder, and 15.5% of agar, pH 7.0).

### Microorganism isolation and identification

To search for strains with keratinase properties, soil samples were collected from various regions of Kazakhstan. Strain T7 was isolated from soil near the city of Taraz in the southern part of Kazakhstan, located at coordinates 42.9° N, 71.36667° E. During the isolation of the strain, the ability of proteolytic and keratinolytic microorganisms to hydrolyze milk proteins, gelatin, and keratin in agarized media was analyzed. For isolation, 1 g of soil was resuspended in 9 mL of 0.9% (w/v) NaCl, and the suspension was serially diluted 10-fold to obtain the pure culture. The 1000-fold dilution was used for seeding on the agarized medium: 100 μl was cultured on a plate with nutrient agar at 37°C for 48 h. Proteolytic and keratinolytic activities of the pure strain were tested by inoculation on plates with skim milk, gelatin, and feather agar. Transparent zones around the colonies confirmed the corresponding enzymatic activity of the strain. The purity of the colonies was checked by Gram staining and light microscopy. The strain was identified by sequencing of a conserved DNA locus according to [10, 21]. For the sequencing, genomic DNA of the strain was isolated using the Genomic Wizard Purification Kit (Promega, Madison, USA). The 16s ribosomal-RNA gene fragment was amplified by PCR with universal primers 27F and 1492R. The amplicon was sequenced by the Sanger method [24] with BigDye™ Terminator v3.1 (Thermo Fisher Scientific, USA). DNA fragments were separated on an ABI 3730xl automated sequencer (Applied Biosystems, USA). Chromatograms were analyzed and compared with the reference sequence using the Vector NTI software package version 11 and the NCBI database (http://blast.ncbi.nlm.nih.gov/Blast.cgi).

### Enzyme extract preparation

The enzyme extract was prepared as described in ref. [10]. Briefly, cells of the strain were cultured in 5 mL of LB broth for 16 h at 37°C with shaking at 170 rpm in a shaker. The culture was inoculated into 150 mL of feather broth supplemented with 2% of yeast extract. Culturing was carried out for 48 h under the same conditions. The supernatant was clarified by centrifugation at 10 000 × *g* at 4°C for 10 min, filtered through a 0.22 μm pore size membrane to remove microparticles and bacterial cells, and was used in experiments.

### Determination of enzymatic activity

Keratinase activity was determined according to [25] with keratin azure (St. Louis, MO, USA) as a substrate. Proteolytic activity was determined according to [26] using azocasein (St. Louis, MO, USA) as a substrate. Determination of α-amylase activity was performed in 100 mM phosphate buffer pH 6.0 at 80°C by the method of reducing sugars adapted to potato starch (Sigma-Aldrich, St. Louis, MO, USA) as a substrate [27]. Alkaline phosphatase activity was assayed according to [23] using *p*-nitrophenyl phosphate disodium salt 6-hydrate (PanReac-AppliChem, Darmstadt, Germany) as a substrate in 100 mM phosphate buffer pH 10.3. Esterase activity was determined at 40°C in 100 mM phosphate buffer pH 7.0 according to [22] by means of 4-nitrophenyl octanoate (Thermo Fisher, Kandel, Germany) as a substrate.

### Effects of temperature and pH on enzymatic activity

The effect of temperature and pH on enzymatic activity was evaluated as previously described [10]. The effect of temperature on keratinase activity was assessed by a reaction in the temperature range of 30–80°C (with intervals of 10°C). The maximum enzymatic activity was set to 100%. For thermostability assays, the enzyme extract was preincubated for 2 h at 50, 60, or 70°C in 0.1 M Tris buffer (pH 8.5), and then the residual activity was measured at 60°C in 0.1 M Tris-HCl buffer (pH 8.5). Results were expressed as a percentage relative to the activity of the extract not subjected to thermal incubation, which was set to 100%. The enzymatic activity was measured within a pH range of 3.0 to 11.0. The following buffer systems were employed: citrate buffer (pH 3.0–6.0), sodium phosphate buffer (pH 6.0–7.5), Tris-HCl buffer (pH 7.5–9.0), and glycine-NaOH buffer (pH 9.0–11.0). The obtained values were converted to relative units, assuming the maximum value to be 100%. For pH stability experiments, the enzyme extract was preincubated for 3 h in 0.1 M citrate buffer (pH 6.0), 0.1 M 8.0 Tris-HCl buffer (pH 8.0), or 0.1 M glycine-NaOH buffer (pH 10.0), and then residual activity was measured in 0.2 M Tris-HCl buffer (pH 8.5). The results were expressed as a percentage relative to the activity of the nonpreincubated extract, which was set to 100%.

### Zymography

Zymographic analysis was performed according to a published protocol [25]. The ability of the enzyme extract to degrade various protein substrates was evaluated by SDS-PAGE in a 4–10% gel copolymerized with (w/v) casein (0.1%), gelatin (1%), or keratin (0.7%). The enzyme extract was added to SDS-PAGE sample buffer (125 mM Tris-HCl pH 6.8, 0.002% of bromophenol blue, 4% of SDS, and 20% of glycerol) in a ratio (sample:buffer) of 4:6. PMSF (5 mM), EDTA (5 mM), Pepstatin A (0.035 mM), and E64 (0.01 mM) were added as inhibitors of serine, metalloprotease, asparagine, and cysteine proteases, respectively. Samples were not boiled, and no β-mercaptoethanol or DTT was added before electrophoresis. Gels were washed for 10 min twice in 2.5% Triton X-100 and incubated in 500 mM Tris-HCl (pH 8.5) at 50°С for 20 h. The gels were stained with 0.08% Coomassie Brilliant Blue G-250 (AppliChem, Darmstadt, Germany) in 20% ethanol with 1.6% of orthophosphoric acid and 8% of ammonium sulfate for 4 h and clarified in MilliQ water for 3–4 h until the hydrolysis zones got completely clarified. Protein markers (New England Biolabs, cat. # P7719S) were used to estimate molecular weight.

### Hydrolysis of protein substrates

Hydrolysis of protein substrates was carried out according to a previously described method [10]. Gelatin, keratin, casein, and BSA were tested as substrates for hydrolysis by the enzyme extract. The selection of these substrates is due to their classification into different classes of proteins (phosphoproteins, fibrous proteins, and globular proteins). Each substrate was dissolved in 1 mL of 50 mM Tris-HCl (pH 8.5) to a concentration of 1 mg/mL and treated with the enzyme extract at 60°C for 30 min with periodic sampling for analysis. The substrate without enzyme extract treatment served as a control. Hydrolysis products were analyzed by SDS-PAGE in a 12% gel. Gels were stained with 0.08% Coomassie Brilliant Blue R-250 (AppliChem, Darmstadt, Germany) in 50% ethanol with 10% of acetic acid. Protein markers (New England Biolabs cat. # P7719S) were utilized for molecular-weight determination.

### Hydrolysis of keratin raw materials

The hydrolysis of the feathers was carried out as previously described [10]. The ability of *B*. *paralicheniformis* strain T7 to degrade chicken and goose feathers was investigated next. A culture of *B*. *paralicheniformis* strain T7 was grown in 15 mL of nutrient broth in a shaker incubator at 37°C and 170 rpm for 18 h. After that, 1 mL of the inoculum was added to 0.075 g of chicken and goose feathers in glass test tubes containing 10 mL of sodium phosphate buffer (pH 7.0). The degradation of samples was carried out in a shaker incubator at 37°C and 250 rpm for 4 days. The degree of degradation of samples was assessed by measurement of the dry weight of unhydrolyzed samples. For this purpose, the culture was passed through pre-weighed filter paper, and the residue was washed twice with distilled water and dried at 60°C to a constant weight. The result was expressed as a percentage of the original weight of the samples, which was set to 100%. Feathers prepared in the same way but without the culture served as a control.

### SEM of the products of feather hydrolysis

The chicken and goose feather were inoculated with 1 mL of the sterile enzyme extract in 10 mL of 0.1 M Tris-HCl buffer (pH 8.5) supplemented with 0.02% of sodium azide. Incubation was carried out for 2–7 days at 37°C. To evaluate the effect of the *B*. *paralicheniformis* T7 bacterium on the degradation process, hydrolysis with the unfiltered enzyme extract without sodium azide was performed under the same conditions. Samples under the same conditions but without the enzyme extract were used as controls. The feathers were collected and fixed in 0.2 M cacodylate buffer (pH 7.0) containing 1% of glutaraldehyde at 4°C for 6 h. The moisture in each sample was replaced by ethanol, and this step was repeated six times. The samples were then dried with a K-850 critical point dryer (Quorum Technologist Ltd) and coated with gold by means of an Eiko IB-5 ion coater. SEM of the samples was carried out according to [28]. The samples were placed on stubs and gold-sputtered (10 nm). An Auriga Crossbeam 540 (Carl Zeiss, Jena, Germany) scanning electron microscope operating at 3 kV captured the images.

### Identification of extracellular enzymes by mass-spectrometric analysis

The annotated genome of *B*. *paralicheniformis* T7 (GenBank accession number CP124861) was employed as a guide to create a library of all the proteins in the strain. For mass-spectrometric analysis of the secretory proteome, the enzyme extract was concentrated 150-fold on a Pierce^TM^ Protein Concentrator (10K MWCO: 10 kDa molecular weight cutoff). Protein separation was performed by SDS-PAGE in a 12% gel. Proteins were extracted from the gel and treated with trypsin (Promega, Madison, WI, USA) to fragment them into peptides. The peptides were separated on an Acclaim Pep-Map RSLC column (Thermo Scientific, Waltham, MA, USA) in an acetonitrile gradient. An unmodified CaptiveSpray ion source was employed to interface the HPLC system with a Maxis Impact II Instrument (Bruker, Germany). The mass range of the MS scan was set to m/z 150–2200 in positive ion polarity mode. *B*. *paralicheniformis* T7 extracellular proteases were identified on the Mascot platform with the help of the amino acid sequence library of this strain.

### Fermentation by *B*. *paralicheniformis* T7 in a bioreactor and obtaining of an enzyme preparation

A 10-liter Biostat bioreactor (Sartorius, Germany) was used to test *B*. *paralicheniformis* T7’s ability to produce proteolytic enzymes under submerged-fermentation conditions. Submerged fermentation was carried out in accordance with [29]. A single colony was inoculated into 5 mL of nutrient broth and cultured at 37°C in a shaker incubator at 180 rpm for 18 h. The grown culture was transferred into 200 mL of feather medium supplemented with 0.2% of yeast extract and grown under the same conditions for 24 h. The culture was inoculated into 6 L of a sterile feather medium with 0.2% of yeast extract in a 10-L fermenter. The conditions for submerged fermentation were as follows: temperature: 37°C, agitation: 450 rpm, aeration: 6–10 L/min, and culturing time: 48 h. Samples for determination of the number of colony-forming units and measurement of keratinase activity were taken periodically.

The strain’s culture was cleared of cells and substrate residues by centrifugation at 11,000 × *g* and sterilized by microfiltration on a UPIRO-018 bench filtration apparatus (Vladisart, Vladimir, Russia) using membrane polyethersulfone module MKM46020 (Vladisart, Vladimir, Russia) with a cutoff of 0.22 μm. Then, 150 mL of the sterile culture was concentrated by evaporation for 1 h on an RV05 basic rotary evaporator (IKA-Werke, Staufen, Germany) at 55°C via 50 rpm rotation in vacuum (4.9 kPa) until a volume of 3 mL was reached. After that, 450 mL of sterile culture was frozen at −80°С in a U570 Ultra low freezer (New Brunswick Scientific, Enfield, CT, USA) and dried in a BETA 2–8 LDplus (Christ, Osterode, Germany) at −90°С in vacuum (0.028 mBar) for 48 h. The lyophilized enzyme preparation was ground into a powder. The remaining sterile culture was spray dried on an OM-1500A spray dryer (Shang Hai Ou Meng, China) under the following conditions: chamber inlet temperature 55°C, chamber outlet temperature 40°C, spray pulse duration 1 s, and extract delivery rate 4.5 mL/min. Enzymatic activity was measured in the resulting samples.

### Software tools, bioinformatics, and statistical analysis

Chromatograms after sequencing were analyzed in Vector NTI Advance 11. The Mascot platform was used for mass-spectrometric protein identification. Online resource Peptide Signal IP 5.0 (http://www.cbs.dtu.dk/services/SignalP/) was employed for signal peptide prediction. All activity assays were performed three times independently. Mean values and standard deviations (SD) were calculated in GraphPad Prism version 8.0.1. Enzymatic activity is presented as the mean ± standard deviation (n = 3).

## Results

### Isolation and identification of the proteolytic strain *B*. *paralicheniformis* T7

**Fig 1:**
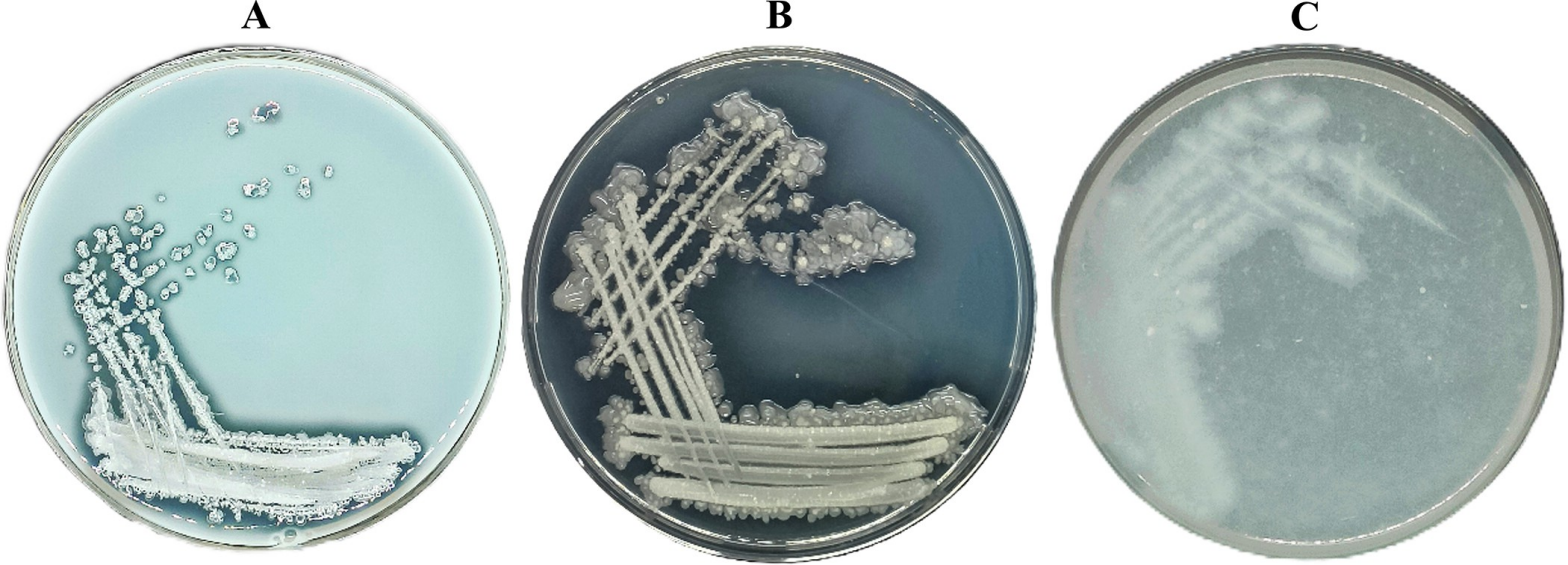
The Т7 strain on skim milk (A), gelatin (B) and feather (C) agar after 24 h incubation at 37°C.

Isolate T7 was isolated from soil samples collected near Taraz city. The strain demonstrated proteolytic and keratinolytic properties on milk and feather agar (Fig 1). On nutrient agar, isolate T7 formed irregularly shaped colonies having turbid and opaque areas with a shiny surface, 2–4 mm in diameter. When isolate T7 was cultured in nutrient broth for 24 h, it demonstrated moderate growth. Isolate T7 is capable of growth at 37–55°C and pH of the medium 5.0–9.0.

Taxonomic analysis and morphological examination of colonies showed that isolate T7 forms irregularly shaped colonies with turbid and opaque areas, milky in color, 2–4 mm in diameter, and a glossy surface. When isolate T7 was cultured in nutrient broth for 24 h, it showed moderately rapid growth. During the growth, the medium became turbid, and bacterial flocs arose in the culture medium. A hard-to-break film formed at the liquid–air interface.

A Gram-staining smear showed gram-positive rod-shaped bacteria, which are typical of bacteria from the genus *Bacillus*. Both stand-alone and chained bacterial cells were found. Sequencing of 16s rRNA by the Sanger method indicated that strain T7 belongs to the species *B*. *paralicheniformis* with identity 100%.

### Effects of temperature and pH on the enzymatic activity of the extract

**Fig 2:**
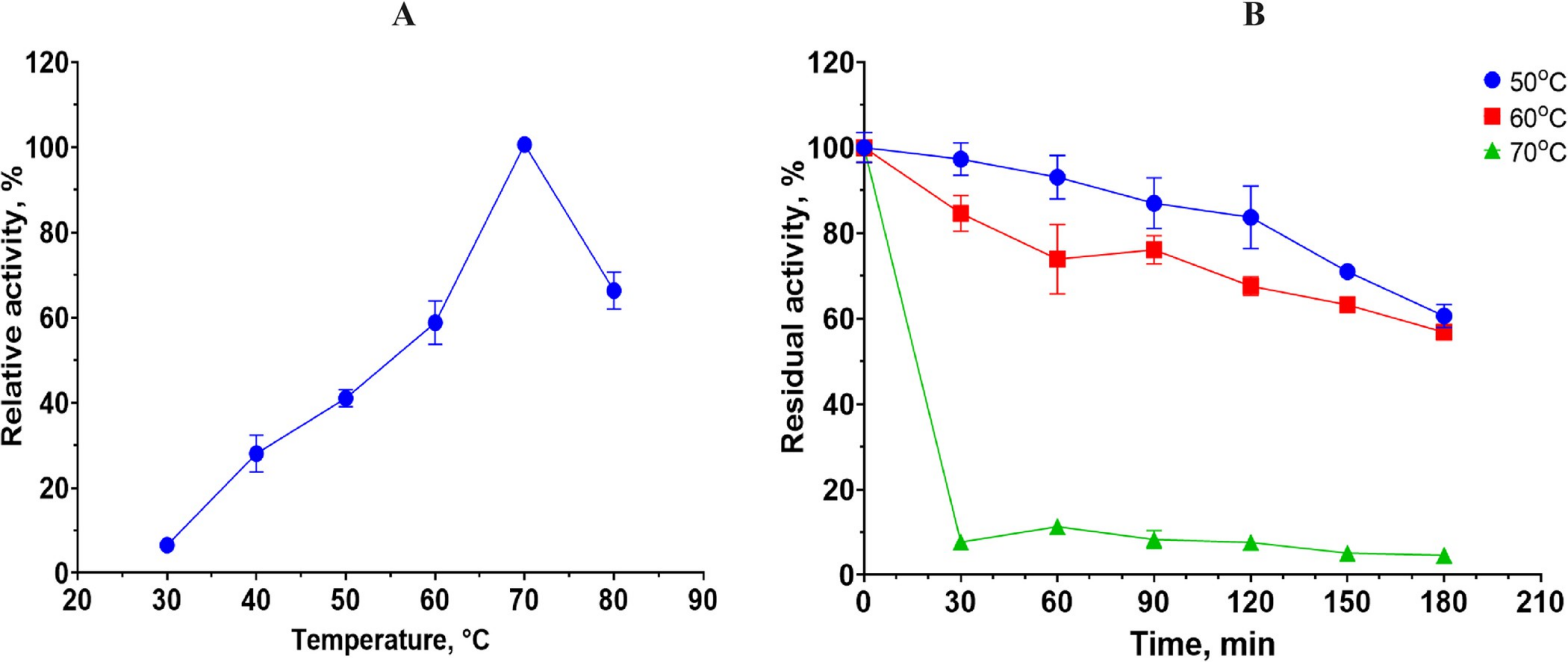
A: The impact of temperature on keratinase activity of the *B*. *paralicheniformis* T7 enzyme extract. B: The impact of temperature, at 50, 60, or 70°C, on the stability of the *B*. *paralicheniformis* T7 enzyme extract.

The dependence of the *B*. *paralicheniformis* T7 enzyme extract’s activity on temperature in the range of 30–80°C was studied on keratin azure. The extract manifested the highest keratinase activity at 70°C (Fig 2). The keratinases in the extract retained 60% activity in the range of 40–70°C.

The results of the thermostability assay of the enzyme extract during preincubation at 50, 60, or 70°C for 3 h are presented in Fig 2B.

**Fig 3:**
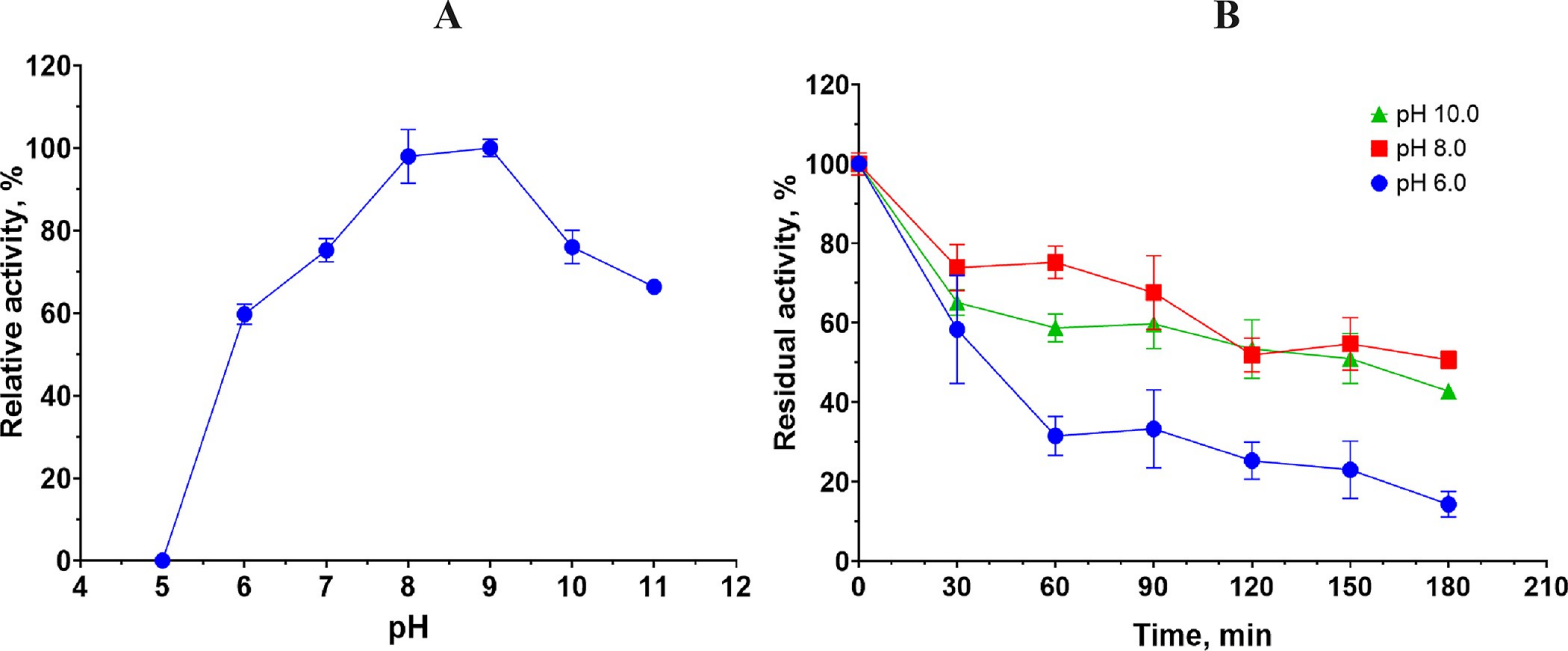
A: The impact of pH on keratinase activity in the *B*. *paralicheniformis* T7 enzyme extract. B: The impact of pH at 6.0, 8.0, and 10.0 on the stability of *B*. *paralicheniformis* T7 enzyme extract.

The dependence of the keratinase activity of the enzyme extract from *B*. *paralicheniformis* T7 on pH was studied in the range of 4–10. The data showed that the keratinases in the extract were active across a wide pH range and retained more than 60% of their activity at pH 6.0–10.0. The optimum was achieved in Tris-HCl buffer with pH 9.0 (Fig 3A). The results on the pH stability of the enzyme extract during preincubation at pH 6.0, 8.0, or 10.0 for 3 h are shown in Fig 3B.

### Substrate specificity

**Fig 4:**
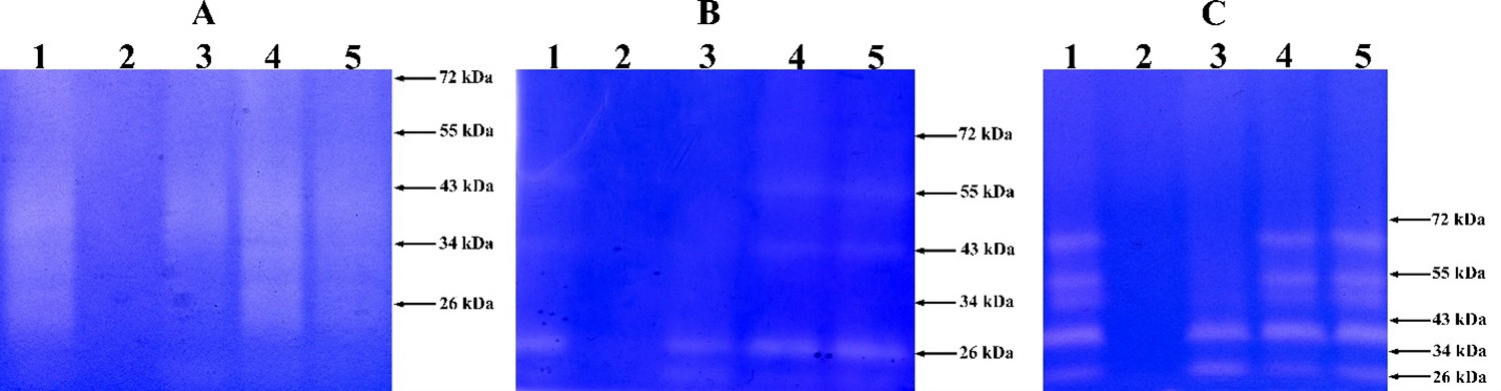
A zymogram with copolymerized keratin (A), casein (B), or gelatin (C) for the enzyme extract from *B*. *paralicheniformis* T7. Enzyme extract (lane 1), enzyme extract with PMSF (lane 2), enzyme extract with EDTA (lane 3), enzyme extract with Pepstatin A (lane 4), and enzyme extract with E64 (lane 5).

Zymography revealed that the enzyme extract of *B*. *paralicheniformis* T7 contained proteases that hydrolyze casein, keratin, and gelatin (Fig 4). PMSF inhibited the activity of the enzyme extract from *B*. *paralicheniformis* T7 in reactions with any types of substrates; therefore, it can be concluded that most of the enzymes contained in the enzyme extract belong to the class of serine proteases. In the zymographic analysis on casein and gelatin, three enzymes were also inhibited by EDTA, indicating that they belong to the class of metalloproteases.

The presence of several brightening zones in all zymograms indicated that the enzyme extract is a cocktail of proteases with different molecular masses.

### Hydrolysis of casein, gelatin, BSA, and keratin

**Fig 5:**
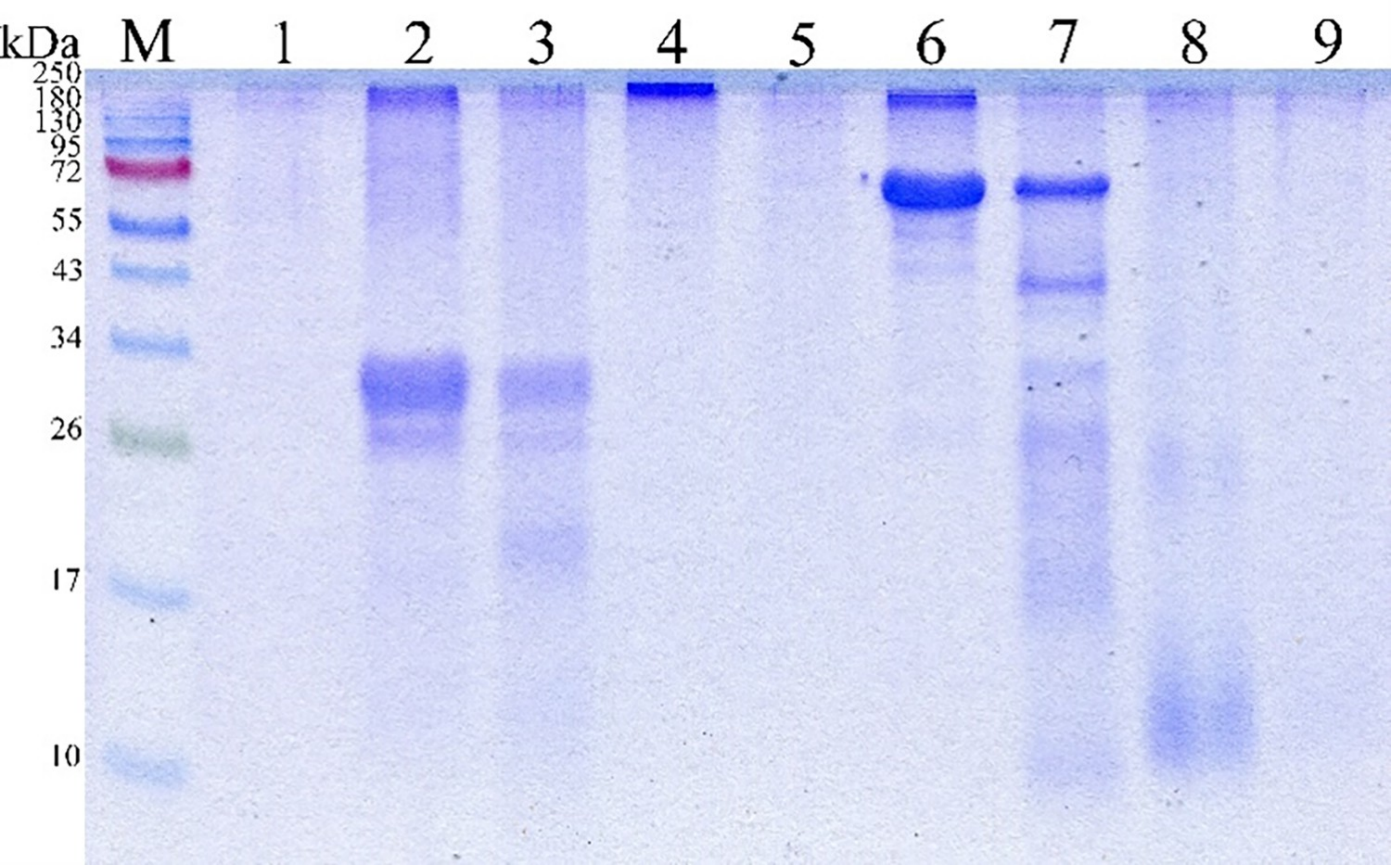
Hydrolysis of various proteins by the enzyme extract from *B*. *paralicheniformis* T7. М: protein markers (NEB cat. # P7719S); 1: enzyme extract; 2: casein; 3: hydrolyzed casein in 15 s; 4: gelatin; 5: hydrolyzed gelatin in 5 min; 6: BSA; 7: hydrolyzed BSA in 5 min; 8: keratin; 9: hydrolyzed keratin in 30 min.

The rate of hydrolysis is of great importance for the cleavage of proteins and depends on the nature of a substrate. Different proteins like casein, gelatin, BSA, and keratin were selected to study the rate of hydrolysis. Hydrolysis of each substrate was performed at 60°C in 0.1 M Tris-HCl buffer pH 8.5. At the end of the experiment, electrophoretic separation of hydrolysis products by SDS-PAGE was conducted (Fig 5).

The hydrolysis by the keratinases of *B*. *paralicheniformis* strain T7 was found to differ significantly in speed among the substrates. Casein proved to be hydrolyzed faster than the other substrates (Fig 5); casein hydrolysis occurred after 15 s of incubation. One minute was sufficient to hydrolyze ovalbumin; BSA, hemoglobin, and gelatin got hydrolyzed within 5 min. Feather keratin was the most resistant to the hydrolytic action of *B*. *paralicheniformis* T7 enzymes; its hydrolysis took 30 min.

### Degradation of chicken and goose feathers

**Fig 6:**
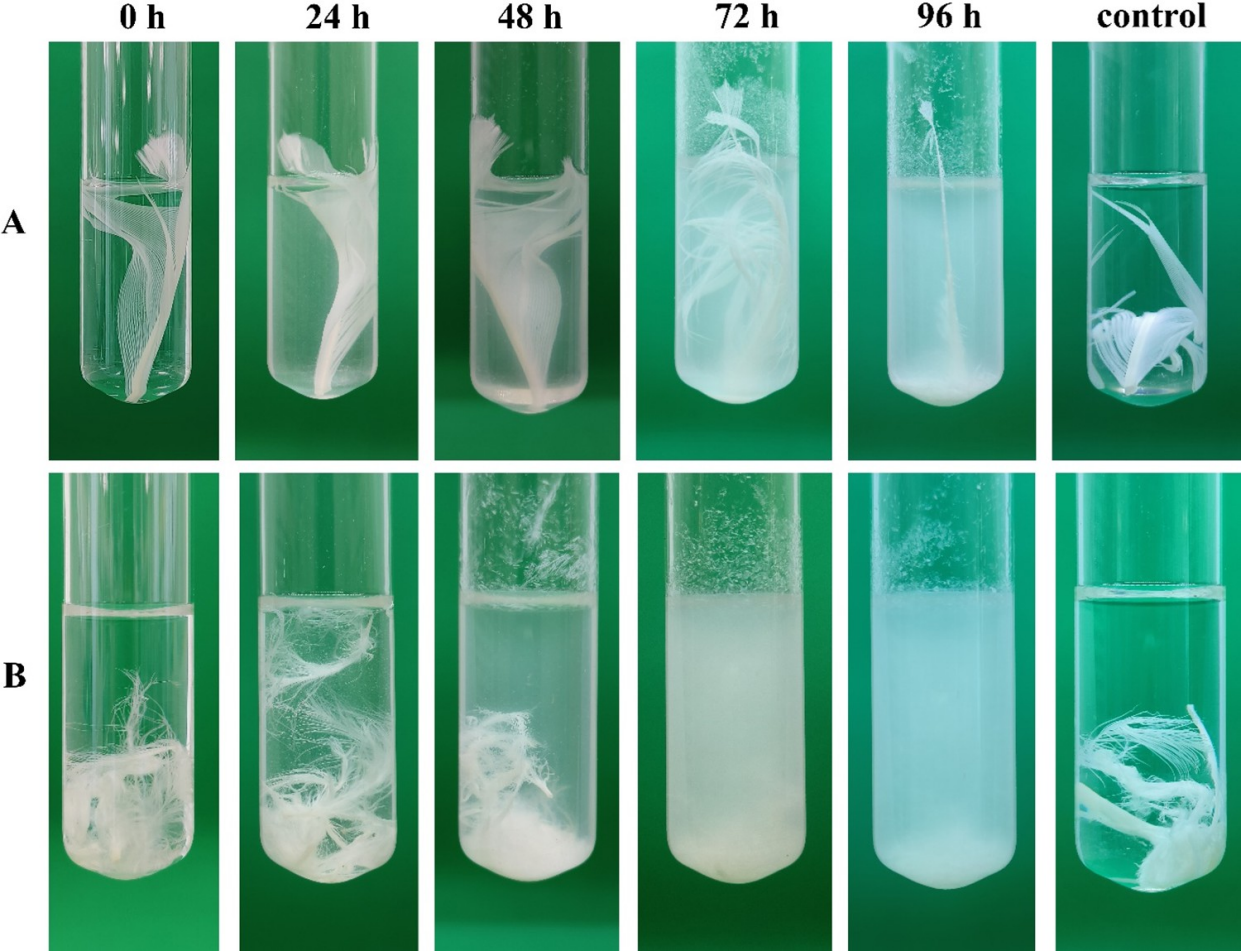
Degradation of goose (A) and chicken (B) feathers by *B*. *paralicheniformis* T7 culture for 96 h. Control: A control sample after 96 h of incubation without the addition of the bacterial culture.

Treatment of chicken and goose feathers with *B*. *paralicheniformis* T7 revealed that this strain has degradative properties toward both types of feathers. Fig 6 shows the dynamics of feather degradation.

Measurements of chicken and goose feather degradation suggested that chicken feathers degraded 100% after 4 days of incubation with *B*. *paralicheniformis* T7. Goose feathers under the same conditions and after the same period degraded by 60%. Measurements of keratinase activity on keratin azure after culturing on chicken and goose feathers gave 57.3 ± 2.8 and 73.7 ± 8.4 U/mL, respectively. The protease activity of the culture fluid toward azocasein was 620.5 ± 10.0 and 804.7 ± 10.5 U/mL, respectively.

### SEM

**Fig 7:**
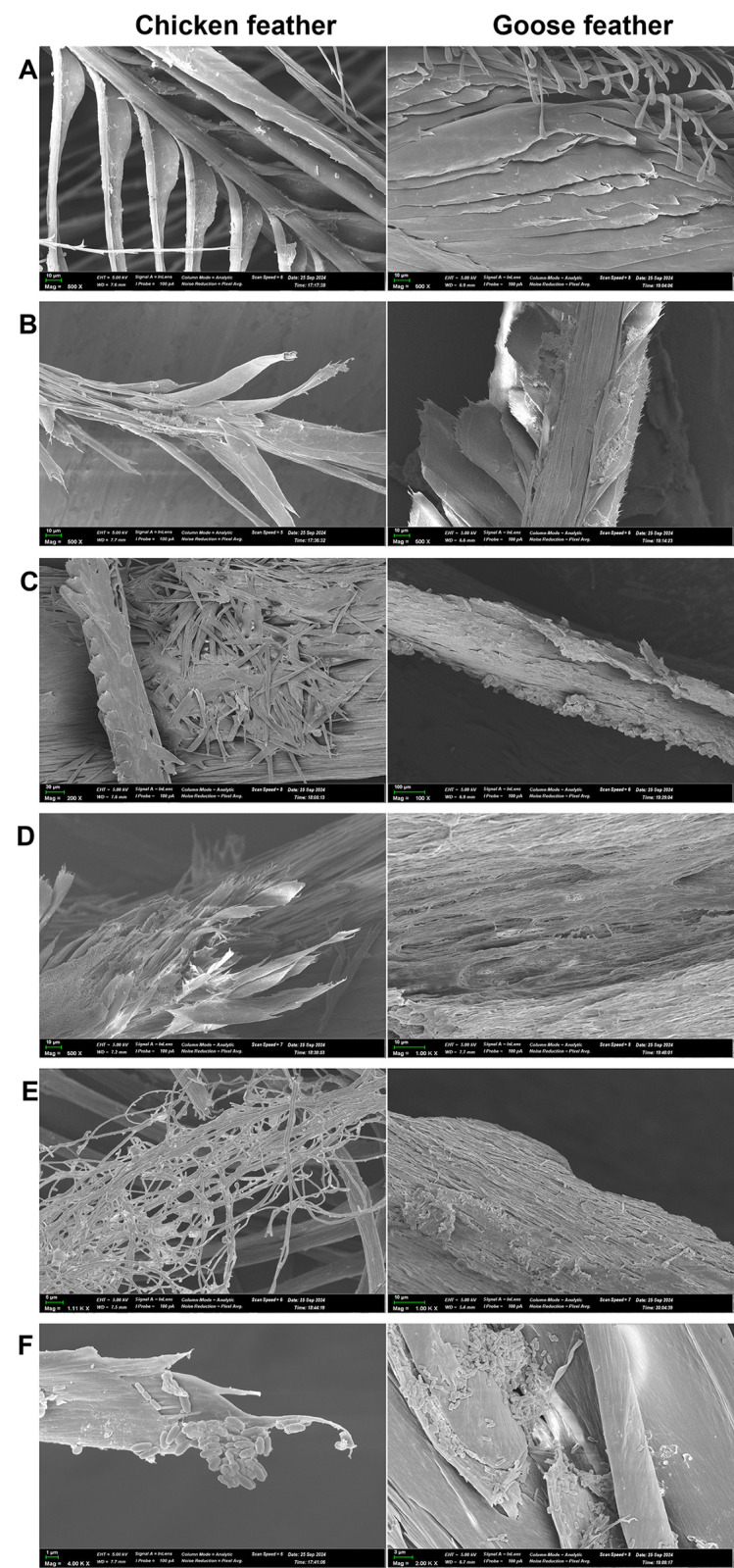
An SEM image of a chicken feather after hydrolysis by *B*. *paralicheniformis* T7 strain, where (A) is an intact feather; (B–E) the feathers after 24, 48, 72, and 96 h of incubation, respectively; (F) the feathers after 24 h incubation (magnification 2000–4000×).

Changes in the microstructure of chicken and goose feathers were examined under a scanning electron microscope during degradation by *B*. *paralicheniformis* T7 enzymes. Fig 7 presents photographs of a feather subjected to the hydrolysis. Feather stems with barbs and barbules were clearly visible in the images of untreated feathers (Fig 7A), and after 24–96 h, their degradation was observed (Fig 7B–7E). The photographs with high magnification show that cells of *B*. *paralicheniformis* strain T7 adhered to the feather surface (Fig 7F).

Table 1 shows the results of measuring keratinase activity in the supernatant obtained by incubating chicken and goose feathers with *B*. *paralicheniformis* T7 culture for 96 h.

**Table 1:** Keratinase activity of samples with hydrolyzed chicken and goose feathers, depending on the incubation period.

Feather	Keratinase activity depending on incubation time, U/mL				
	0 h	24 h	48 h	72 h	96 h
Chicken	0	33.5 ± 3.8	54.3 ± 1.0	36.3 ± 6.4	25.6 ± 3.4
Goose	0	73.1 ± 1.8	52.1 ± 2.4	50.7 ± 1.6	17.4 ± 1.1

### Mass-spectrometric analysis of the secretory proteome

Seven peptidases with molecular masses of 154.8, 86.2, 41.2, 53.3, 60.6, 30.5, and 26.8 kDa were identified in the enzyme extract by HPLC-Q/TOF mass spectrometry and Mascot analysis against all 4388 protein-coding sequences of *B*. *paralicheniformis* T7 (Table 2). In addition to peptidases, the following hydrolases were detectable: α-amylase (55.2 kDa), phosphatase (59.8 kDa), and lipase (26.8 kDa).

**Table 2:** Proteases identified by protein mass spectrometry and proteomic analysis in the enzyme extract of the *B*. *paralicheniformis* T7 strain (GenBank accession number CP124861.1).

Molecular weight, kDa	Protein	DNA Locus	Protein length (amino acid residues)	Signal peptide
154.8	S8 family serine peptidase	2407118..2411416	1432	32
86.2	S8 family serine peptidase	120102..122522	806	28
41.2	S8 family serine peptidase	2934598..2935733	378	29
53.3	S41 family peptidase	1794133..1795523	463	36
60.6	M14 family metallocarboxypeptidase	3290537..3292180	547	28
30.5	M55 family metallopeptidase	2645411..2646235	274	-
26.8	M42 family metallopeptidase	2945002..2945730	242	-
55.2	Alpha-amylase AmyS	3447405..3448942	483	29
59.8	Alkaline Phosphatase	1500803..1502464	525	28
26.8	GDSL-type esterase/lipase family protein	1322691..1323403	238	11

### Fermentation by *B*. *paralicheniformis* T7 strain in a bioreactor and obtaining of the proteolytic preparation

**Fig 8:**
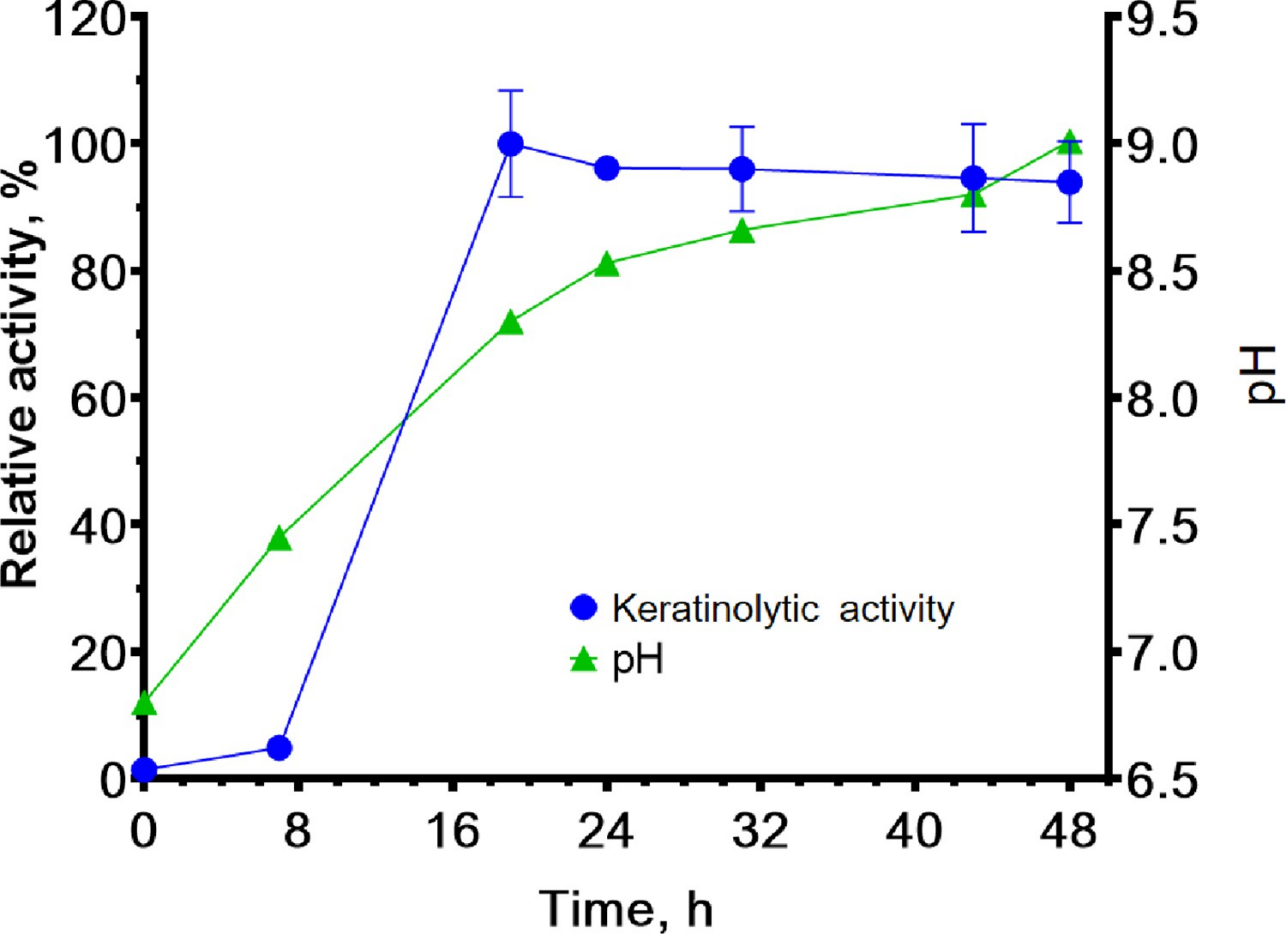
Pilot scale production of keratinases by *B*. *paralicheniformis* T7.

To obtain proteolytic enzymes by the biotechnological method, the *B*. *paralicheniformis* T7 strain was cultivated by submerged fermentation in a 10-L bioreactor. After 18, 24, and 48 h of the fermentation, the density of the culture was 0.3 × 10^9^, 1 × 10^9^, and 5.3 × 10^9^ colony-forming units (CFU)/mL, respectively. Fig 8 shows the dependence of keratinase activity and pH in the culture on fermentation duration in the bioreactor. After the fermentation, the keratinase activity in the culture fluid toward keratin azure was 63.6 ± 5.8 U/mL. The protease activity against azocasein was 715.7 ± 40.2 U/mL.

The following techniques were tested to obtain an enzyme preparation from the culture liquid after fermentation by *B*. *paralicheniformis* T7 strain: concentration by vacuum evaporation at 55°C for 1 h, lyophilization at −52°C under vacuum for 63 h, and spray-drying in an air stream at 55°C. By evaporation under reduced pressure, the enzyme extract was concentrated 50-fold. The keratinase, protease, α-amylase, phosphatase, and esterase/lipase activities in the obtained preparations were measured and are presented in Table 3.

**Table 3:** Activity of the enzyme extract after concentration, freeze-drying, or spray-drying.

Activity	Concentrate enzyme extract, U/mL	Lyophile powder, U/g	Spray Drier powder, U/g
Keratinase	2,656.7 ± 170.4	57,733.3 ± 8,911.4	53,466.7 ± 757.2
Protease	29,886.7 ± 642.9	567,066.7 ± 4,822.2	585,333.3 ± 4,277.1
Amylase	176.1 ± 16.3	2,823.0 ± 266.8	2,395.8 ± 893.7
Phosphatase	23.9 ± 1.8	364.2 ± 74.8	416.7 ± 52.4
Esterase/Lipase	510.9 ± 12.2	17,618. ± 610.3	15,328.1 ± 528.6

## Discussion

Microbial proteases are in demand in various fields of human activity: food, feed, and processing industries; production of detergents; and the pharmaceutical industry. The cost of microbial enzymes is substantially lower than that of plant and eukaryotic analogs. Microorganisms produce enzymes faster than mammalian and plant cells do; this enzyme production does not depend on climatic conditions or seasonal changes and does not violate ethical or environmental standards [30]. Depending on substrate specificity, keratinases (EC 3.4.21/24/99) capable of hydrolyzing tough fibrillar proteins (keratin and collagen) can be distinguished among proteases [31]. Keratinases are known to efficiently cleave both keratin-rich materials such as feathers, nails, and wool and soluble protein substrates such as casein, whey proteins, and BSA [32]. Feather waste from poultry farming appears to be a promising source of cheap protein raw materials because feathers account for ~5–7% of the total weight of a chicken. The high enzymatic activity of keratinases allows them to hydrolyze very resistant keratins, and for this reason, they are considered the most promising proteolytic enzymes in the industry [33, 34].

*Bacillus* bacteria contain an efficient enzymatic system, including a variety of proteolytic enzymes, which allows them to occupy different ecological niches [30]. *Bacillus* species are found everywhere, including various types of soil, compost, and landfills. They are predominantly mesophilic microorganisms, but at the same time, a number of their enzymes have high thermostability [35, 36]. Good producer properties of bacteria of the genus *Bacillus* have enabled this taxon to occupy a dominant position in industrial microbiology [37]. Among microorganisms that degrade keratin, *Bacillus* species are well-known producers of keratinases [19, 38–41]; many of these microbes have been isolated from soil and poultry waste [10, 42].

Strain T7 was isolated from soil and identified as *B*. *paralicheniformis* on the basis of cultivation-related, morphological, proteomic, molecular genetic, and genomic features. Studies suggest that this strain, when cultured on feather agar, produces keratinolytic enzymes that hydrolyze feather keratin, thereby forming characteristic clear zones. *B*. *paralicheniformis* T7 strain is able to grow on a feather medium with added salts in which feather keratin is the only source of organic matter.

Biochemical characteristics of the enzyme extract of *B*. *paralicheniformis* T7 on keratin azure were investigated in this work. The findings show that the strain’s keratinases work best at pH 9.0. Our comparison with previously described proteases revealed that most of *Bacillus* keratinases are also alkaline (Table 4), with the exception of the keratinase from *B*. *licheniformis* dcs1 [43]. The keratinases from *B*. *paralicheniformis* T7 manifested the highest activity at 70°C. Comparative Table 4 indicates that among the bacillary keratinases, the maximum activity is mainly observed at 50–60°C; only enzymes from *Bacillus* sp. CSK2 and *Bacillus* sp. NFH5 exceed this value [11, 41].

**Table 4:** Maximum temperatures and pH optima of various bacillary keratinases.

Strain	Maximum temperature (°C)	pH optimum	Reference
*B*. *paralicheniformis* T7	70	9.0	This work
*Bacillus* sp. A5.3	60	8.5	[10]
*B*. *pumilus* AR57	45	9.0	[44]
*B*. *subtilis* NRC3	50	7.5	[45]
*B*. *licheniformis* PWD-1	50	7.5	[46]
*B*. *subtilis* KD-N2	55	8.5	[40]
*B*. *licheniformis* RPk	60	9.0	[47]
*B*. *subtilis* MA21	60	9.0	[48]
*B*. *zhangzhouensis* BK111	60	9.5	[49]
*B*. *subtilis* 1271	40–50	10.0	[39]
*B*. *licheniformis* 1269	40–50	10.0	[39]
*B*. *cereus* 1268	40–50	10.0	[39]
*B*. *halodurans* PPKS-2	60	11.0	[50]
*Bacillus* sp. CSK2	60–80	8.0	[41]
*B*. *cereus* YQ15	60	10.0	[17]
*B*. *subtilis* SCK6	60	10.0	[38]
*B*. *licheniformis* dcs1	45	7.0	[43]
*Bacillus* sp. Nnolim-K1	60	8.0	[51]
*Bacillus* sp. NFH5	90	8.0	[11]
*Bacillus* sp. BK111	60	9.5	[49]

The assay of thermostability of the enzyme extract showed that the keratinases contained in the extract have high thermostability. The keratinases withstood one-hour preincubation at 50°C with 93% of the original activity retained. Preincubation at 60°C reduced residual activity by 26% over the same period. The keratinases were less stable after preincubation at 70°C; in this case, the residual keratinase activity of the extract was 11%. Two-hour preincubation at 50, 60, or 70°C diminished the activity by 16%, 32%, and 92%, respectively. Nonetheless, even after 3 h of preincubation, the keratinases retained residual activity at 60% and 56% after the 50 and 60°C incubation, respectively. The thermostability of enzymes from *B*. *paralicheniformis* T7 is higher than that of *Bacillus* sp. A.5.3 [10]. Keratinases from *B*. *paralicheniformis* T7 are more thermostable than previously characterized enzymes from *B*. *velezensis* Y1, which lose more than 80% of activity after 2 h of preincubation at 55°C and after 10 min at 60°C [52]. Three-hour preincubation under alkaline conditions at pH 8.0 and 10.0 reduces the activity of strain keratinases by no more than 50–60%. Keratinases are more sensitive to acidic conditions; preincubation in citrate buffer with pH 6.0 for 3 h diminished keratinase activity by more than 85%.

Zymographic analyses on protein substrates in the presence of the protease inhibitors PMSF, EDTA, Pepstatin A, or E64 suggest that the keratinase and protease activities in the enzyme extract are predominantly provided by serine proteases and metalloproteases. It has been observed earlier that the proteolytic activity of the enzyme extract of *B*. *subtilis* A5.3 and of other *Bacillus* representatives is strongly inhibited by PMSF [39, 42, 50, 53–55], and a negative effect of EDTA has also been reported for proteases from *B*. *halodurans* PPKS-2 [50], *Bacillus* sp. MKR5 [56], and *Chryseobacterium* sp. kr6 [57]. Our zymographic analyses were performed on casein, keratin, and gelatin, which belong to different structural classes of proteins. The results showed hydrolysis of these proteins by proteases with different molecular weights. Similar zymographic data have been obtained with extracts from *B*. *subtilis* SCL, *B*. *subtilis* 1271, and *Bacillus* sp. A5.3 [10, 39, 58]. More proteases are involved in the hydrolysis of gelatin: five enzymes.

Hydrolysis of the five different protein substrates showed that milk casein is the most susceptible to this hydrolysis: 1 mg of casein is hydrolyzed in 15 s. BSA and gelatin are more resistant to the action of *B*. *paralicheniformis* T7 proteases: their hydrolysis took 5 min. Keratinases of this strain hydrolyzed 1 mg of keratin in 30 min. In a comparison of our results with those from *Bacillus* sp. A5.3, *B*. *paralicheniformis* strain T7 turned out to be comparable in keratinolytic activity but superior in proteolytic activity. *B*. *paralicheniformis* T7 enzymes took much less time to hydrolyze casein, and BSA than did proteases from *Bacillus* sp. A5.3 [10]. Casein, BSA, gelatin, and keratin are the main proteins of milk, of blood plasma, and of waste from meat-processing plants and poultry farms. The information on their sensitivity to proteolytic enzymes of *B*. *paralicheniformis* T7 is relevant for practical application.

The enzymes of *B*. *paralicheniformis* T7 are characterized by high hydrolytic activity against keratin. It is known that α- and β-keratins, which are components of feathers, have high mechanical strength and are resistant to the action of most proteases [59]; hydrolysis of these keratins requires a synergistic action of enzymes [1]. In our work, chicken feathers were less resistant to the hydrolytic action of the strain than goose feathers were. After 2 days, degradation of hooks and beards was observed, and by the end of the fourth day, a chicken feather was hydrolyzed completely. A comparison with *B*. *subtilis* strain A5.3 regarding the ability to break down keratin revealed that *B*. *paralicheniformis* T7 breaks down feathers by 120 h faster than *B*. *subtilis* strain A5.3 does [10].

A comparative analysis of protease and keratinase activities of the *B*. *paralicheniformis* T7 extract after cultivation of the microbe on different substrates showed that there is a difference in the activity of the culture liquid after cultivation on chicken and after cultivation on goose feathers. A comparison of keratinase activity after 96 h of cultivation of the strain on chicken and goose feathers suggested that goose feathers stimulate keratinase activity by 28% better than chicken feathers do. The same was found for protease activity; the difference in protease activity was 29%.

SEM of hydrolyzed chicken and goose feathers showed that their hydrolysis proceeds much more efficiently when bacterial culture is used rather than the enzyme extract alone. SEM detected the adhesion of *B*. *paralicheniformis* T7 bacterial cells to the feather surface. More intense degradation of the feathers was noted at the sites of cell attachment. Similar results are reported in refs. [1, 10, 58]. As for *B*. *subtilis* A5.3 and *B*. *licheniformis* RG1 strains, the extracellular keratinolytic activity of *B*. *licheniformis* RG1 without live bacterial cells also does not result in complete feather degradation [1]. Accordingly, it can be said that the adhesion of bacterial cells to the substrate surface plays an important role in efficient and rapid hydrolysis of keratin-containing raw materials. Daily measurement of activity in chicken and goose feather samples as a result of their incubation with *B*. *paralicheniformis* T7 showed that keratinase activity in goose feather samples reached a maximum earlier than in chicken feather samples: after 24 and 48 h of incubation, respectively. Further incubation led to a decrease in keratinase activity in both samples. In the goose feather supernatant, the maximum keratinase activity was 73.1 ± 1.8 U/mL, while in the chicken feather supernatant, the maximum activity was 54.3 ± 1.0 U/mL. The differences in keratinase activity levels can be explained by dissimilarity of structure between chicken and goose feathers, as evidenced by microphotographs in Fig 7.

Although the detailed mechanism of keratin hydrolysis has not been fully elucidated [60], the general theory of keratin degradation implies prior breakage of disulfide bonds to allow keratinases access to keratin polypeptide chains [33]. Cysteine dioxygenase, glutathione reductase, thioredoxin reductase, dihydrolipoyl dehydrogenase, peptide methionine sulfoxide reductase, and other reductases can catalyze disulfide bond breakage [61]. The main enzymes involved in keratin degradation are keratinolytic endopeptidases (from families S1, S8, S16, M4, M16, and M36), exopeptidases (from families S9, S10, M14, M28, M38, and M55), and oligopeptidases (from families M3 and M32) [60]. After disulfide bonds are severed, keratinolytic endopeptidases hydrolyze keratin chains to form peptides, which are subjected to the hydrolytic action of keratinolytic exopeptidases, which degrade peptides at both ends, and oligopeptidases cleave oligopeptides into individual amino acids [62]. From the analysis of the *B*. *paralicheniformis* T7 genome, it follows that the thioredoxin-disulfide reductase detected in the genome (accession # CP124861.1, DNA Locus 430718..431668) may take part in the disulfide bond degradation with the participation of the enzymes of this strain, and the endo- and exopeptidases detected in the enzymatic extract of the strain are involved in the further degradation of keratins.

Our mass-spectrometric analysis of the secretory proteome detected seven proteases. Four of them belong to serine peptidase families S8 and S41, and the other three to metallopeptidase families M14, M42, and M55. Serine proteases of the S8 family have in common the presence of the Asp-His-Ser triad. His plays a dual role of a proton acceptor and donor at different reaction steps, while Asp positions the His residue in the correct orientation to facilitate a nucleophilic attack by Ser [63]. These proteases have high keratinolytic specificity; for example, the keratinolytic/caseinolytic activity ratio of the keratinase KerK from *B*. *amyloliquefaciens* belonging to the S8 family is 4.76 [64]. The high keratinolytic activity of S8 family enzymes explains why half of all characterized keratinases are affiliated with this family [60]. Metalloproteases of the M14 family are characterized by the His-Xaa-Xaa-Xaa-Glu motif. Most of metallopeptidases of the M14 family—being carboxypeptidases (and thus showing high regiospecificity toward a carboxyl group)—cleave an amino acid residue from the C terminus [65]. The M55 family of metalloproteases is known to activate under starvation conditions, thereby providing the cell with peptides as degradation products [66]. This process occurred when *B*. *paralicheniformis* T7 cells were cultured on the minimal feather medium. The role of proteases of the S41 and M42 families in the degradation of keratin by the *B*. *paralicheniformis* T7 extract has not yet been elucidated, necessitating further investigation of this issue.

Previously, genes of 11 serine proteases from the S8 family have been found in the DNA of *B*. *paralicheniformis* MKU3 by genomic analysis [67]. Two extracellular serine proteases and two metalloproteases have been identified in *B*. *paralicheniformis* MKU3 by LC-MS/MS and proteomic methods. In the secretory proteome of *B*. *paralicheniformis* T7, four serine proteases and three metalloproteases were detected here. It is noteworthy that two proteases from *B*. *paralicheniformis* MKU3 with molecular masses of 87 and 60 kDa correspond to the S8 family serine peptidase and the M14 family metallocarboxypeptidase detected in the secretory proteome of *B*. *paralicheniformis* T7. A literature analysis revealed that S8 family serine proteases play an important role in the hydrolysis of feather keratin. For instance, the high activities of a serine peptidase/subtilisin-like enzyme of the S8 family from *B*. *licheniformis* KRLr [68] and peptidase KerT (also belonging to the S8 family) from *Thermoactinomyces* sp. YT06 [69] toward keratin have been detected. Heat-stable keratinase from *Meiothermus taiwanensis* WR-220 [70], keratinase from *B*. *paralicheniformis* RPk [47], and alkaline keratinase from *B*. *amyloliquefaciens* K11 [71] are also affiliated with the subtilisin family of proteases. Surfactant-stable keratinase from *Brevibacillus parabrevis* CGMCC 10798 is also a member of the serine protease family [72]. Besides serine proteases, metalloproteases are also involved in keratin hydrolysis, e.g., alkaline metalloprotease from *Bacillus* sp. Okoh-K1 [73]. Fungal keratinases are mainly represented by serine and metalloproteases too [74, 75]. A minireview [74] indicates that out of 14 keratinases from nonpathogenic fungi, eight enzymes are serine proteases (five keratinases from the S8 family and three serine proteases from an unidentified family), three are metalloproteases (two enzymes from the M28 family and one enzyme from the M3 family), and one is asparagine protease.

The analysis showed that all identified serine peptidases, M14 metallocarboxypeptidase, α-amylase, phosphatase, and esterase from *B*. *paralicheniformis* T7 have signal peptides of length 11–36 amino acid residues. In addition to peptidases, α-amylase AmyS, alkaline phosphatase, and esterase were identified in the enzyme extract.

Submerged fermentation of the minimal feather medium by *B*. *paralicheniformis* strain T7 in a bioreactor revealed that the strain efficiently produces keratinases after 7 h of fermentation, and the keratinase activity reaches its maximum after 19 h of fermentation. In comparison, the *B*. *subtilis* S14 strain reaches its maximum keratinase activity after 23 h of fermentation [76], *B*. *subtilis* strain 8 after 48 h [77], and *Pseudomonas aeruginosa* strain YK17 after 72 h [78]. The feather medium is a cheap and useful substrate for obtaining proteolytic enzymes. Adding yeast extract or peptone to the main substrate improves production properties of keratinase strains [79].

Given that the keratinases of *B*. *paralicheniformis* T7 are thermostable enzymes, aside from lyophilization, an attempt to obtain concentrated keratinase preparations was made here by vacuum evaporation and spray drying in heated air. Keratinase activities in the concentrate, lyophilizate, and dry powder were 2,656.7 ± 170.4 U/mL, 57,733.3 ± 8,911.4 U/g, and 53,466.7 ± 757.2 U/g, respectively. Additionally, other types of hydrolase activities were measured: protease activity on azocasein, α-amylase activity on starch, phosphatase activity on *p*-nitrophenyl phosphate, and esterase activity on *p*-nitrophenyl octanoate. The protease activity in the concentrate, lyophilizate, and dry powder was an order of magnitude higher than keratinase activity and was 29,886.7 ± 642.9 U/mL, 567,066.7 ± 4,822.2 U/g, and 585,333.3 ± 4,277.1 U/g, respectively. *Bacillus* strains are known for their amylolytic activity [80]. A number of *Bacillus* α-amylases are thermostable and active at 60–80°C [81]. *B*. *paralicheniformis* T7 proved to be no exception, the enzyme preparation obtained by 1 h vacuum evaporation at 60°C showed significant α-amylase activity at 80°C: 176.1 ± 16.3 U/mL, implying thermostability of the α-amylase. In the lyophilized and dried preparations, α-amylase activity was 2,823.0 ± 266.8 and 2,395.8 ± 893.7 U/g, respectively. Alkaline phosphatase also exhibited high thermostability, and after 1 h of concentration at 60°C, it had an activity of 23.9 ± 1.8 U/mL, 364.2 ± 74.8 U/g, and 416.7 ± 52.4 U/g in the lyophilized and dried preparations. It is likely that the biosynthesis of alkaline phosphatase can go up after the fermentation conditions are optimized, as is the case for *B*. *paralicheniformis* strain APSO [23]. Closely related *B*. *paralicheniformis* strains are known to possess lipase activity, which is a type of esterase activity similar to that of *B*. *licheniformis* NCU CS-5 [82]. The esterase activity of *B*. *paralicheniformis* strain T7 on *p*-nitrophenyl octanoate was 510.9 ± 12.2 U/mL, 17,618.0 ± 610.3 U/g, and 15,328.1 ± 528.6 U/g for the concentrated, lyophilized, and dried preparations, respectively, pointing to good potential of this strain as a microbial esterase and lipase producer.

*B*. *paralicheniformis* T7 has many favorable characteristics: the strain is not demanding in terms of nutrient-medium composition; it can grow either on rich media (nutrient broth or LB broth) or on minimal media (feather broth). The strain is not sensitive to temperature conditions: grows at 37–55°C and pH 5.0–9.0. *B*. *paralicheniformis* T7 does not need any activators for enzyme production, and the capacity of the strain for submerged fermentation makes it promising for practical use.

The enzyme cocktail from *B*. *paralicheniformis* T7 may be applied practically as an enzyme additive for poultry feed, in hydrolysis of keratin-containing waste of slaughterhouses (blood, horns, hooves, hides, and putti joints), and/or in utilization of feather waste of poultry farms. The amylolytic activity of the *B*. *paralicheniformis* T7 enzyme cocktail can be utilized in the enzymatic hydrolysis of starch-containing raw materials, while its lipase activity can aid in the processing of fats and oils. High caseinolytic and phosphatase activities point to a possible application of the enzyme cocktail as a milk-curdling agent for milk processing and cheese-making technologies. The advantages of the enzymes from the extract from *B*. *paralicheniformis* T7 include their resistance to moderately high temperatures (50–60°C), their activity in a wide pH range (6.0–11.0), and the absence of substrate limitations. The enzymes of the cocktail hydrolyze fibrillar proteins (keratin and collagen), phosphoproteins (casein), globular proteins (albumin), polysaccharides (starch), and fats. The availability of commercial keratinases is limited due to low productivity and such properties as thermostability and activity in a narrow pH range [83], and therefore the search for new sources of thermostable keratinases seems relevant. The proteolytic enzymes of *B*. *paralicheniformis* T7, which have high hydrolytic activity and good thermal stability and are active at acidic, neutral, and alkaline pH values, should be of interest to the feed, tannery, and detergent industries. High-temperature starch processing requires thermostable α-amylases [83], and the α-amylase of *B*. *paralicheniformis* T7, active at 80°C, appears promising for this procedure. Its unique lipase (triacylglycerol acyl hydrolase) catalyzes hydrolysis, esterification, and alcoholysis reactions [84]. The lipase of *B*. *paralicheniformis* T7 can fulfill the needs of various industries, such as those producing biodiesel, food and beverages, leather, textiles, detergents, pharmaceuticals, and medical goods. Further research on *B*. *paralicheniformis* T7 will focus on characterizing each enzyme from the extract. The information obtained by LC-MS/MS and genomic sequencing will provide individual recombinant proteins for more detailed biochemical studies as well as allow the role of each enzyme in substrate hydrolysis to be assessed.

Thus, our experiments underscore high potential of *B*. *paralicheniformis* strain T7 as a producer of enzymes with keratinase, protease, amylase, phosphatase, and lipase activities. Further research will focus on these activities in more detail and on selecting fermentation conditions for this strain to increase its production capacity.

## Conclusion

This study describes a soil strain of *B*. *paralicheniformis*, T7, that has high keratinase activity. The enzymes secreted by the strain include thermostable alkaline keratinases belonging to serine peptidase and metallopeptidase families. Culturing of *B*. *paralicheniformis* T7 on chicken feathers showed that the strain completely degrades them in 4 days. SEM images suggest that *B*. *paralicheniformis* T7 cells stick to the feather surface very strongly, which speeds up the breakdown process. Proteomic analyses identified seven peptidases as well as α-amylase, phosphatase, and esterase/lipase in the extract of the secreted matter. Keratinase activity was maximal after 19 h of submerged fermentation of feathers by *B*. *paralicheniformis* T7. Concentrated preparations with high keratinase, protease, collagenase, amylase, phosphatase, and lipase activities were obtained from the extract of the strain. The findings point to promising potential of *B*. *paralicheniformis* strain T7 as a producer of thermostable keratinases and other hydrolytic enzymes.
